# Supplementary material for: Five-year pediatric use of a digital wearable fitness device: lessons from a pilot case study
Source: JAMIA Open. 2021 Aug 2;4(3):ooab054. doi: 10.1093/jamiaopen/ooab054 (PMC8327370; doi:10.1093/jamiaopen/ooab054)
Supplement: ooab054_Supplementary_Data [file ooab054_supplementary_data.zip › supplementary methods.docx]

# Supplementary Methods

Fitbit allows user/programmers to download raw level device data by first registering through their Application Programming Interface (API). While the methodology to access Fitbit data has changed over the years, the current API enables all data (even going back several years) to be easily downloaded into a statistical programming environment, such as R. No specific Fitbit R packages are needed.

Developers should first visit https://dev.fitbit.com/apps/new and login with their usual user-level Fitbit credentials. They can then register a new application using https://dev.fitbit.com/apps/new. An application name and short description need to be entered. An institutional name, website, terms of service, and privacy policy URLs also need to be entered. Under OAuth 2.0 Application Type, “Server” should be selected, though the other types could possibly also work. Read-only access should be chosen. Any HTTPS URL can be specified under the Callback URL, such as https://www.google.com. Click Register to create the new application. Fitbit then provides two key secret pieces of information. The OAuth 2.0 Client ID (e.g. “21D3TH”) and Client Secret (e.g. “85f611859cb9a92c71d4920a51064554”) are now specific to this application, and both will be used to gain access to secured Fitbit data.

From the application detail page (e.g. https://dev.fitbit.com/apps/details/21D3TH, which will specific for the Client ID), click on the “OAuth 2.0 tutorial page” link. Most of the details on the resulting form will already be filled out. The Selected Scopes should all be selected already. Click on the automatically generated authorization URL in the middle of the page. A new browser window will appear to login. This time, the login is to specify whose data is now about to be accessed. After successful login, the browser will be viewing the Callback URL, except the URL has an additional code added (e.g. continuing the above example, https://www.google.com/?code=1a0533...6cbf406#_=_). This is now the authorization code, and it must now be exchanged for an access token. Copy only the code portion from the URL (e.g. “1a0533...6cbf406”), return to the OAuth 2.0 tutorial page, and paste it into the appropriate code field.

Another command is then automatically created (e.g. “curl -I -X POST \...”). Copy this entire and execute it at a terminal or command line (e.g. Mac OS Terminal). The output will resemble something like {"access_token":"eyJhbGc…4d3gtSxw-4OmRt…UI","expires_in":28800,"refresh_token":"b661…e1e64","scope":"sleep activity weight social location profile heartrate settings nutrition","token_type":"Bearer","user_id":"21D3TH "}. If the default settings were used, this access token is now usable for 8 hours.

The actual data is now retrievable through additional curl commands, either performed at the terminal prompt or using RCurl in R. For example, these commands will download the step data between January 9, 2013 and April 18, 2013 (using the above generated access token):

require(RCurl)

h = basicTextGatherer()

curlPerform(url = "https://api.fitbit.com/1.2/user/-/activities/steps/date/2013-01-09/2013-04-18.json", httpheader=c(Authorization=”eyJhbGc…4d3gtSxw-4OmRt…UI”), writefunction = h$update)

From experience, downloading blocks of data spanning no larger than 100 days (and then loop/iterating to cover a larger time frame with 100-day blocks) is a very predictable way to download a large batch of data.

More information on the Fitbit API is provided here: https://dev.fitbit.com/build/reference/web-api/oauth2/

Sample code implementing these instructions is available at the Github repository: https://github.com/kimibutte/Fitbit-Data
