# Supplementary figures and images for: Five-year pediatric use of a digital wearable fitness device: lessons from a pilot case study
Source: JAMIA Open. 2021 Aug 2;4(3):ooab054. doi: 10.1093/jamiaopen/ooab054 (PMC8327370; doi:10.1093/jamiaopen/ooab054)

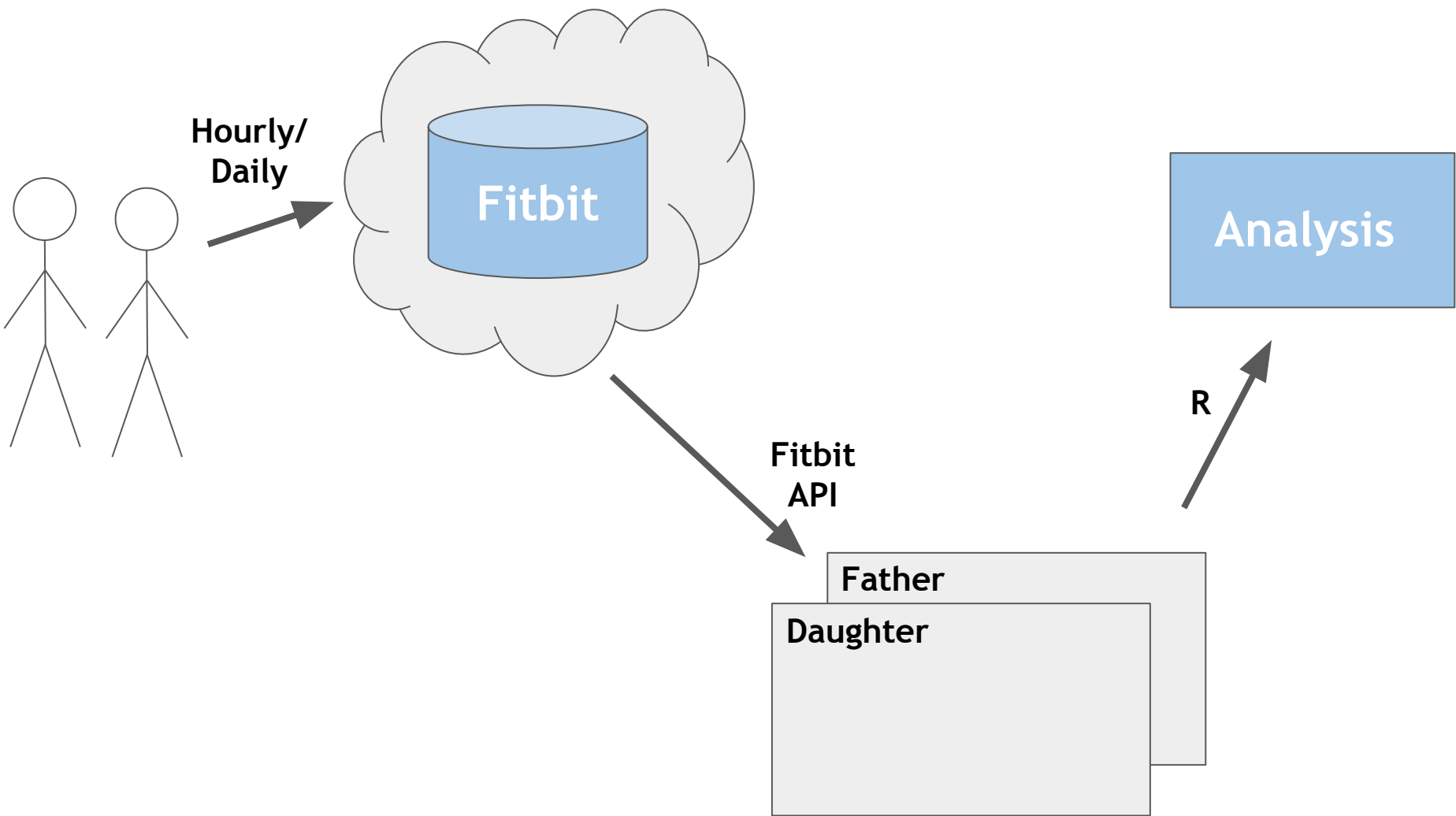

Supplement: ooab054_Supplementary_Data [file ooab054_supplementary_data.zip › supplementary figure 1.pdf]
